# Novel Targets for Stroke Therapy: Special Focus on TRPC Channels and TRPC6

**DOI:** 10.3389/fnagi.2020.00070

**Published:** 2020-03-18

**Authors:** Lu Liu, Lijuan Gu, Manli Chen, Yueying Zheng, Xiaoxing Xiong, Shengmei Zhu

**Affiliations:** ^1^Department of Anesthesiology, The First Affiliated Hospital, College of Medicine, Zhejiang University, Hangzhou, China; ^2^Central Laboratory, Renmin Hospital of Wuhan University, Wuhan, China

**Keywords:** TRP ion channels, TRPCs, TRPC6 channel, neuronal survival, ischemic stroke

## Abstract

Stroke remains a leading cause of death, disability, and medical care burden worldwide. However, transformation from laboratory findings toward effective pharmacological interventions for clinical stroke has been unsatisfactory. Novel evidence has been gained on the underlying mechanisms and therapeutic potential related to the transient receptor potential (TRP) channels in several disorders. The TRP superfamily consists of a diverse group of Ca^2+^ permeable non-selective cation channels. In particular, the members of TRP subfamilies, TRP canonical (TRPC) channels and TRPC6, have been found in different cell types in the whole body and have high levels of expression in the central nervous system (CNS). Notably, the TRPCs and TRPC6 channel have been implicated in neurite outgrowth and neuronal survival during normal development and in a range of CNS pathological conditions. Recent studies have shown that suppression of TRPC6 channel degradation prevents ischemic neuronal cell death in experimental stroke. Accumulating evidence supports the important functions of TRPC6 in brain ischemia. We have highlighted some crucial advancement that points toward an important involvement of TRPCs and TRPC6 in ischemic stroke. This review will make an overview of the TRP and TRPC channels due to their roles as targets for clinical trials and CNS disorders. Besides, the primary goal is to discuss and update the critical role of TRPC6 channels in stroke and provide a promising target for stroke prevention and therapy.

## Introduction

Ischemic stroke is induced by the obstruction of an artery or multiple arteries leading to the brain. Focal impairment or occlusion of blood circulation to the brain impairs the normal function of neurons. The mechanisms underlying ischemic stroke are complex, and include excitotoxicity, oxidative and nitrosative stress, Ca^2+^ overload, inflammation, and apoptosis (Szydlowska and Tymianski, 2010; Khoshnam et al., 2017). Among these mechanisms, intracellular Ca^2+^ overload remains a vital role in neuronal injury associated with ischemic stroke (Choi, 1995). Glutamate receptors, such as *N*-methyl-D-aspartate receptor (NMDAR), are thought to be major pathways for intracellular Ca^2+^ influx in the central nervous system (CNS) after cerebral ischemia-reperfusion (IR) injury. Excessive NMDARs activation and the following Ca^2+^ influx through NMDARs are crucial steps required for initiating ischemic cell death (Szydlowska and Tymianski, 2010; Lai et al., 2011). To date, pre-clinical studies have provided substantial evidences for the neuroprotective effect of NMDAR antagonists in experimental ischemic stroke (Ginsberg, 2008). However, for several decades, clinical trials of NMDAR antagonists have all ended up with failure to show beneficial effects due to their narrow therapeutic windows and adverse effects (Wu and Tymianski, 2018). Thus, effective therapeutic interventions for ischemic stroke are urgently required.

Despite the pivotal functions of NMDARs, non-glutamate mechanisms have drawn attention as promising Ca^2+^ influx pathways involved in brain ischemia. In this respect, researchers shifted focus toward the transient receptor potential (TRP) channels (Szydlowska and Tymianski, 2010). TRPs are non-selective cationic channels which have key functions in different disorders (Moran, 2018). The TRP canonical (TRPC) subfamily was proved to be extensively distributed in CNS and have important functions in neuronal development (Tai et al., 2009). Understanding of these channels may drive the researchers to make a significant breakthrough in CNS diseases therapy. Recently, growing evidence indicates that TRPC6 channel has been involved in Ca^2+^ homeostasis and shown to participate in the molecular pathophysiology of ischemic stroke. TRPC6 was reported to have an critical role in neuroprotection in both *in vitro* and *in vivo* models of ischemic stroke (Du et al., 2010). In this review, we present a general description of the current understanding of TRPs and TRPC subfamily, with an emphasis on their involvement in clinical trials and CNS dysfunctions. Furthermore, this review concentrates on evidence-based advancements of TRPC6 in CNS disorders and cerebral ischemia. The primary aim is to clarify the relationship between TRPC6 and ischemic stroke and discuss future perspectives.

## The TRP Ion Channel Family

The TRP channels comprise a big family of cation channels that are involved in various physiological and pathological processes. TRPs were first discovered in *Drosophila* in 1960s as a conditional phototransduction mutant (Minke, 1977; Montell et al., 1985). TRPs are commonly distributed in different cell types and tissues, and possess many vital functions in ion homeostasis, sensory transduction, inflammatory responses, innate and adaptive immune responses, and cell survival (Clapham, 2003; Nilius et al., 2007; Ramirez et al., 2018). The channel subunits consist of six transmembrane domains (TDs) that assemble as cation-permeable tetramers (Clapham et al., 2001). However, TRP channels have relatively low selectivity for the transport of cations, such as Na^+^ and Ca^2+^, into the cytoplasm.

The TRPs are divided into seven subfamilies, TRPC (canonical), TRPV (vanilloid), TRPM (melastatin), TRPP (polycystin), TRPML (mucolipin), TRPA (ankyrin), and TRPN (NO-mechano-potential), based on amino acid homology (Nilius et al., 2007). These channels can receive multiple types of intracellular and extracellular information, which in turn can induce a series of different responses. Dysfunctions of these proteins are related to many disorders (Kaneko and Szallasi, 2014); e.g., progressive kidney diseases (TRPC5 and TRPC6) (Winn et al., 2005; Zhou et al., 2017), pulmonary edema (TRPC6) (Weissmann et al., 2012), stroke (TRPC6) (Du et al., 2010), myocardial IR injury (TRPC3/6/7) (He et al., 2017), Huntington’s disease (HD) (TRPC5) (Hong et al., 2015), pruritus (TRPV1,TRPA1) (Moran, 2018), lower urinary tract disorders (TRPV4), pain (TRPV1, TRPA1, TRPM8, and TRPM3), and type 2 diabetes (TRPM5) (Voets et al., 2019), idiopathic rhinitis (TRPA1 and TRPV1) (Van Gerven et al., 2017), irritable bowel syndrome (TRPV1) (Wouters et al., 2016), and genetic diseases (TRPA1, TRPC6, TRPV3/4, TRPM1/4/6, TRPML1, TRPP2) (Moran, 2018).

There have been a number of clinical trials of compounds that regulate TRPV1, TRPV3, TRPV4, TRPA1, and TRPM8 (Moran, 2018). The vanilloid receptor, TRPV1, is identified as an important detector of pain, including heat hyperalgesia, postherpetic neuralgia, and osteoarthritic pain (Moran, 2018). Small molecule antagonists and agonists targeting TRPV1, such as NEO6860, V116517, and capsaicin, have attracted attention in research on multiple pain pathways and have been shown to have clinical potential for use in sustained pain relief (Szallasi et al., 2006; Arendt-Nielsen et al., 2016; Brown et al., 2017; Blair, 2018). However, safety issues, such as impaired noxious heat sensation and hyperthermia, require special consideration. Although several recent clinical trials suggested no increase in body temperature in humans (Arendt-Nielsen et al., 2016; Brown et al., 2017), most TRPV1 antagonists examined previously showed on-target adverse effects (Lee et al., 2017; Manitpisitkul et al., 2018), thus limiting their clinical acceptance. The balance between drug efficacy and side effects in clinical trials remains to be explored. TRPV3 is sensitive to warm temperatures (>33°C) and is predominantly found in skin keratinocytes (Xu et al., 2002; Moqrich et al., 2005). The TRPV3 antagonist, GRC 15300, has successfully finished a phase 1 clinical trial and appears to have potential for pain treatment (Pharmaceuticals, 2018). TRPV3 is also an emerging target for itch and skin diseases in animal models; further studies are required to confirm its utility in treatment of clinical skin diseases. TRPV4 was originally characterized in 2000 as an osmosensor that responds to extracellular osmolarity (Liedtke et al., 2000; Strotmann et al., 2000). TRPV4 performs essential roles in various diseases by regulating Ca^2+^ influx. A clinical trial of TRPV4 blockers and their promising roles in the treatment of dyspnea and pulmonary edema in heart failure and acute decompensated heart failure cases is currently finished in phase 1 stage (GlaxoSmithKline, 2019). Similarly, another single-center study designed to estimate the effects of GSK2798745, a novel TRPV4 inhibitor, on pulmonary gas exchange and pulmonary function in participants with heart failure has already completed a phase 2 clinical trial (GlaxoSmithKline, 2018). TRPA1 has been regarded as a gatekeeper of chronic inflammatory diseases (Bautista et al., 2013). A phase 2a clinical study yielded data indicating that the TRPA1 antagonist, GRC 17536, alleviates pain with no other drug-related side effects in patients with painful diabetic neuropathy, which supports the promising therapeutic efficacy of TRPA1 blockade in pain management (Pharmaceuticals, 2014). PF-05105679 is a TRPM8 antagonist that was shown to reverse cold pain sensation in the cold presser test in humans (Andrews et al., 2015; Gosset et al., 2017). Importantly, blockade of TRPM8 by PF-05105679 showed no effects on core body temperature.

Overall, TRPs have good potential as therapeutic targets for various diseases. Further efforts are still required to explore the specific and detailed functions of TRPs in different disorders.

## The TRPC Subfamily and Their Roles in CNS

The mammalian C-type TRP channels (TRPC proteins) are most closely related to *Drosophila* TRPs. In terms of amino acid similarity, TRPCs can be classified into four subsets: TRPC1, TRPC2, TRPC3/6/7, and TRPC4/5 (Clapham et al., 2001). At a molecular level, all TRPCs consist of a pore–loop motif, three to four NH_2_-terminal ankyrin repeat domains (ARDs), and a COOH-terminal TRP domain (Venkatachalam and Montell, 2007). The ARD is a common protein–protein interaction module, typically consisting of 33 amino acid residues, which forms a canonical helix-loop-helix fold followed by a β-hairpin loop (Islam et al., 2018). ARDs perform crucial roles in various cellular processes, including thetranscription, signal transduction, inflammatory responses, cell development, and cell cycle (Islam et al., 2018). Confocal Förster resonance energy transfer (FRET) measurements revealed that the first ARD in TRPC5 is crucial for homomultimerization (Schindl et al., 2008). ADRs are also closely involved in TRPC4 tetramer assembly (Lepage et al., 2009). In addition, ARDs in TRPC3 in skeletal muscle could mediate TRPC3/1 heteromerization, thus regulating resting cytosolic Ca^2+^ levels (Woo et al., 2014). The novel membrane RING-H2 protein, RNF24, binds with the ARDs of TRPC6 in the Golgi apparatus and results in intracellular retention of TRPC6 without affecting channel activity (Lussier et al., 2008). Thus, ARDs of TRPCs likely participate in their channel heteromerization and trafficking. The TRP domain is a highly conserved sequence of 25 amino acids containing a proline-rich motif and an invariant sequence called the TRP box (Glu-Trp-Lys-Phe-Ala-Arg) (Montell, 2001). A previous study indicated that the TRP domain in TRPC3 is an essential element in regulating the cell response to erythropoietin (Hirschler-Laszkiewicz et al., 2011). The pore region of TRPCs, situated between the fifth and sixth TDs, is associated with an intracellular gate and an extracellular selectivity filter. The conserved LFW motif is a key feature of the TRPC pore loop, which has a pivotal role in hydrophobic interactions and in sustaining the appropriate structure of the ion pore (Li J. et al., 2019).

Transient receptor potential canonical channels are non-selective cation channels with different Ca^2+^ and Na^+^ permeability ratios. TRPCs activation is involved in changes in [Ca^2+^]_*i*_, which governs diverse complex and crucial cellular functions. Evidence suggests that all TRPCs can be commonly activated by phospholipase C (PLC) within numerous stimulation, like inflammatory and IR injury, subsequently mediating Ca^2+^ entry into the cell (Weissmann et al., 2012; Wu et al., 2015). TRPCs are closely connected to PLC activity controlled by modulation of the endogenous agonists diacylglycerols (DAGs) and phosphoinositides. TRPCs were originally identified in sensory nerve endings followed by axons and dendrites of central neurons in the CNS. There have been shown that TRPCs are involved in nerve-growth-cone guidance, neuronal survival, synapse formation, synaptic transmission, and sensory transduction (Li et al., 2005; Jia et al., 2007; Zhou et al., 2008; Quick et al., 2012; Hartmann and Konnerth, 2015). Notably, TPRCs are responsible for inflammation, IR injury, and excitotoxicity (Ramirez et al., 2018).

Alzheimer’s disease (AD) is driven by the cerebral deposition of amyloid-β protein (Aβ), the primary element of the senile plaques seen in the pathological brains. Loss of TRPC1 aggravates memory deficits and cell apoptosis induced by Aβ (Li et al., 2018). It is widely recognized that Aβ generation is closely related to amyloid precursor protein cleavage by γ-secretase. Wang et al. (2015) demonstrated that TRPC6 regulates γ-secretase of amyloid precursor protein. Importantly, a recent clinical case-control research revealed that the TRPC6 mRNA level in peripheral leukocytes is markedly reduced in patients with AD, which provides new insight for clinical diagnosis of AD (Chen et al., 2019). In addition, piperazine, a novel TRPC6 agonist, was shown to decrease long-term potentiation in the 5xFAD mouse, suggesting that piperazine may have potential for AD therapy (Popugaeva et al., 2019). Heteromultimeric channels of TRPC1/4/5 subunits regulate flexible relearning and working memory in mice (Broker-Lai et al., 2017). A study in TRPC5 transgenic mice identified an essential role of TRPC5, activated by G protein-coupled neuronal receptors, in the modulation of innate fear. This result provided strong evidence that TRPC5 channels may be a potential target for the alleviation of fear behavior (Riccio et al., 2009). Previous study has shown that TRPC1 aggravates hippocampal neuronal cell death (Narayanan et al., 2008). A subsequent research suggested that the TRPC1 mediates store-operated Ca^2+^ influx in neurons with mutant Huntingtin protein, suggesting a novel treatment for HD (Wu et al., 2011). Inhibition of TRPC1 is a potentially useful neuroprotective and therapeutic strategy against HD (Wu et al., 2018). In addition, generation of endogenous TRPC5 is increased in both transgenic mice and patients with HD. Knockdown or blockage of TRPC5 promotes neuronal survival and improved neurodegeneration in HD (Hong et al., 2015). The novel TRPC4/5 blocker, M084, was reported to show rapid antidepressant and anxiolytic-like effects in male C57BL/6 mice (Yang et al., 2015). Furthermore, TRPC channels have gained focus on the treatment of ischemic stroke. Decreased TRPC1 expression was shown in ischemic brain tissues. Overexpression of TRPC1 inhibits cerebral I/R injury, whereas TRPC1 knockout has the opposite effects. The underlying mechanism refers to the generation of reactive oxygen species via nicotinamide adenine dinucleotide phosphate (NADPH) oxidase family 4-containing-NADPH oxidase (Xu et al., 2018). Additionally, TRPC6 also holds important roles in stroke therapy, which will be discussed in further detail below.

Taken together, the observations outlined above indicating the importance of TRPCs in CNS disorders suggest that agents targeting TRPCs have great potential for clinical use. While highly selective and specific agonists and antagonists of TRPCs are needed for future study to deeply explore their roles in the treatment of CNS diseases. Even though there is limited evidence, specific channel characters of TRPC channels make them possible to be novel targets for brain ischemia. Therefore, further research efforts are required to find the possible mechanisms underlying the roles of TRPCs in CNS diseases and particularly in ischemic stroke.

## Structure and Characteristics of TRPC6

**FIGURE 1:**
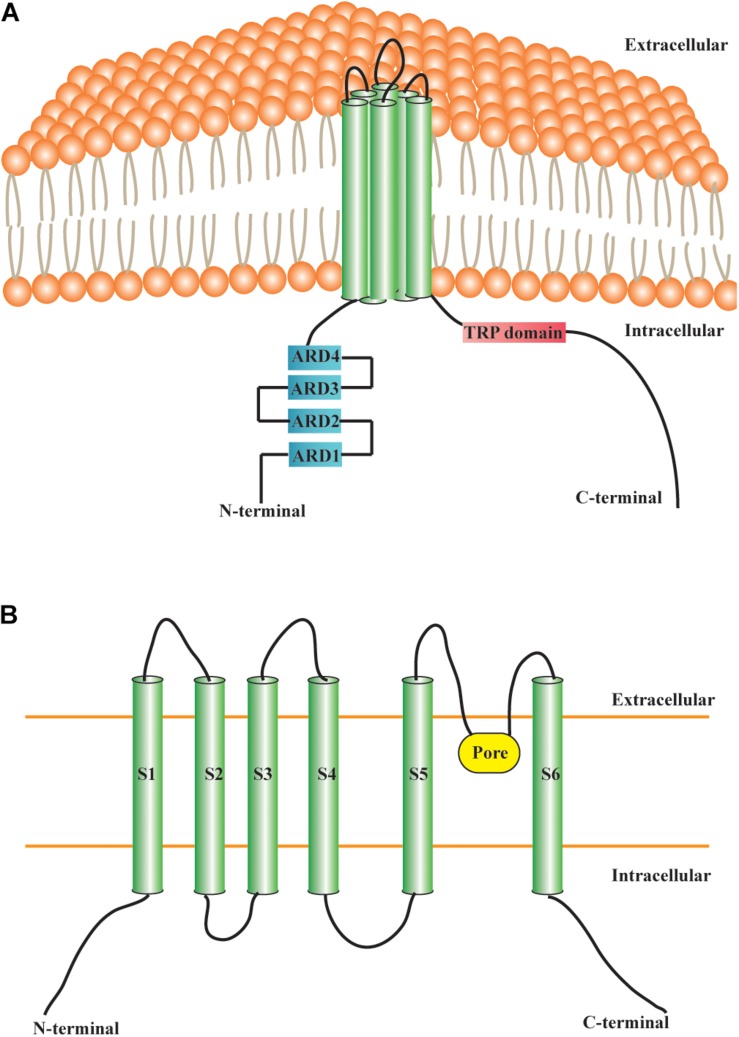
Structural features of TRPC6 channel. **(A)** Transmembrane protein TRPC6 contains six TDs, four NH_2_-terminal ARDs, and a COOH-terminal TRP domain. **(B)** S1–S6 are TDs. The pore region of TRPC6, situated between the S5 and S6, allows the passage of cations. TRP: transient receptor potential; TRPC6: transient receptor potential canonical channel 6; TD: transmembrane domain; ARD: ankyrin repeat domain; S1–S6: S1–S6 are the abbreviations of the first to sixth transmembrane domains.

The TRPC6 channel protein is composed of six TDs, intracellular NH_2_-ARDs, and the COOH-terminal TRPC6 domain (Figure 1). TRPC6 has a molecular weight of approximately 110 kDa and consists of 930 and 931 amino acid residues in mice and humans, respectively. The intracellular cytoplasmic domain and TD are indispensable for TRPC6 assembly (Tang et al., 2018). The TRPC6 channel is permeable to several cations, including Ca^2+^, Na^+^, K^+^, Cs^+^, and Ba^2+^ (Hofmann et al., 1999; Estacion et al., 2006). The relative ion permeability ratio (P_*Ca*__/_P_*Na*_) of TRPC6 is about 5 (Hofmann et al., 1999; Owsianik et al., 2006). Particularly, TRPC6 governs the function and fate of Ca^2+^ homeostasis in various cells and tissues. TRPC6 channel activation in neurons usually occurs in parallel with cytosolic entry of Ca^2+^ ions and the elevation of intracellular Ca^2+^ (Jia et al., 2007; Bouron et al., 2016). TRPC6 is widely expressed in different anatomical regions of the CNS, and is also highly distributed in placenta, lung, pancreas, adipose, heart, kidney, muscle, and other tissues (Riccio et al., 2002). TRPC6 can be activated by DAG, store-depletion, Hyperforin (*St. John’s wort* extract), or H_2_O_2_ (Bouron et al., 2016). DAG is a second messenger that acts as a regulator of key signaling molecules, cellular membrane constituents, and fatty acid metabolism (Massart and Zierath, 2019). It is widely accepted that activation of TRPC6 channels by DAG promotes various cellular responses, although the precise underlying mechanisms remain to be elucidated. By contrast, several studies have indicated that TRPC6 channels could also be store-operated (Yu et al., 2003; El Boustany et al., 2008). Hyperforin has been shown to specifically activate TRPC6 channels, which is mainly responsible for the antidepressant effect of *St. John’s wort* (Yang et al., 2018). There is also a close relation between TRPC6 and reactive oxygen species. H_2_O_2_ was reported to directly activate TRPC6 channels in different cell types (Bouron et al., 2016).

## The Biological Function of TRPC6 in CNS

In the postnatal rat cerebellum, TRPC6 is identified on Purkinje cell bodies, on interneurons in the molecular layer and mature granule cells in the internal granule cell layer, and is required for postnatal cerebellar neuron development (Huang et al., 2007). In cultured cerebellar granule cells, brain-derived neurotrophic factor (BDNF) induces Ca^2+^ elevation and nerve growth-cone extension through TRPC3/6 channels (Li et al., 2005). Besides, TRPC3 and TRPC6 contribute to BDNF-induced cerebellar granule neuron survival (Jia et al., 2007). siRNAs against TRPC3 and TRPC6 reduce BDNF-mediated intracellular Ca^2+^ elevation, neuroprotection, and cAMP-response element binding protein (CREB) activation in cerebellar granule neurons. By contrast, upregulation of TRPC3 or TRPC6 was shown to suppress neuronal apoptosis and promote the transcription of CREB-dependent reporter gene. Additionally, Ca^2+^ influx-dependent TRPC6 channel function is involved in hippocampal neuron dendritic growth via the Ca^2+^/calmodulin-dependent kinase IV (CaMKIV) and CREB pathway (Tai et al., 2008). In particular, the CaMKIV-CREB pathway seems to be a vital downstream molecular mechanism of TRPC6 function in the CNS. Another study showed that TRPC6 is predominantly presented in excitatory synapses and plays important roles in promoting synapse and dendritic spine formation, spatial memory, and learning through the CaMKIV-CREB pathway (Zhou et al., 2008). As an important intracellular second messenger, Ca^2+^ contributes to neuronal development and survival. The interaction between TRPC3/6 channels and the Na^+^/Ca^2+^ exchanger could regulate Ca^2+^ influx in neuronal cells, suggesting that TRPC3/6 may govern the fate of neurons by modulating Ca^2+^ influx (Louhivuori et al., 2010). TRPC6 also acts as a negative regulator that suppresses NMDA-induced Ca^2+^ currents in hippocampal neurons (Shen et al., 2013). Interestingly, NMDAR was shown to regulate transcription and degradation of TRPC6 in neurons in a bidirectional manner through NR2A or NR2B activation (Qu et al., 2017). Hyperforin, a specific agonist of TRPC6, regulates dendritic spine morphology in the hippocampus via TRPC6 activation and increasing intracellular Ca^2+^ transients (Leuner et al., 2013). However, the underlying signaling cascade has not yet been clarified. The same research team also reported the involvement of phosphatidylinositide 3-kinase (PI3K)/protein kinase B (PKB), Ras/mitogen-activated protein kinase/extracellular signal-regulated kinases (Ras/MAPK/ERK), and CaMKIV-CREB pathways (Heiser et al., 2013). Moreover, hyperforin also raises the number of excitatory synapses, dendritic spine density, and dendritic length in experimentally depressed rats by rescuing the decline of TRPC6 expression (Liu et al., 2015). High-throughput sequencing based on TRPC6 in autism spectrum disorder (ASD) patients and controls revealed the presence of more non-synonymous mutations in ASD individuals, suggesting that TRPC6 may carry out as a potential predisposing factor for ASD (Griesi-Oliveira et al., 2015). Furthermore, TRPC3 and TRPC6 could promote normal sensory mechanotransduction in touch and hearing neurons, and therefore knocking out both TRPC3 and TRPC6 resulted in deficits in sensory conduction (Quick et al., 2012). TRPC6 and TRPC7 channels also function in phototransduction in murine retinal ganglion cells (Jiang et al., 2018).

In general, TRPC6 channels are extensively expressed in the CNS and have various crucial functions. Therefore, agents targeting TRPC6 may have potential for the cure of brain disorders.

## TRPC6 and Ischemic Stroke

**FIGURE 2:**
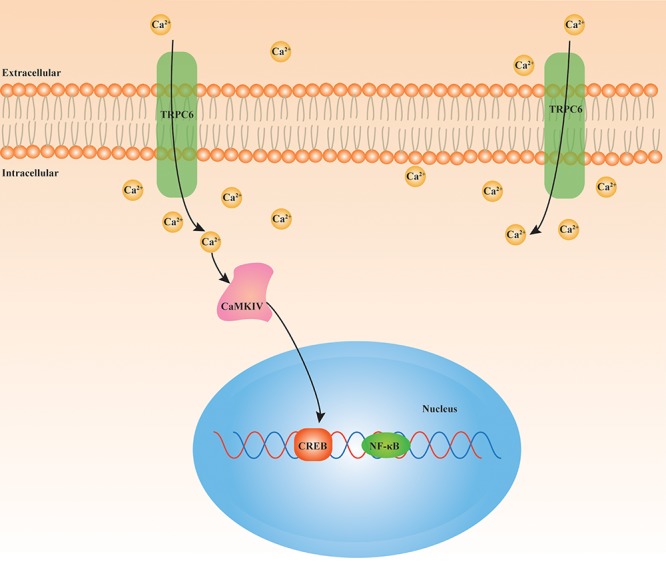
Underlying signaling cascade of TRPC6 in ischemic stroke. TRPC6/Ca^2+^/CAMKIV/CREB pathway is closely involved in the pathophysiological mechanism of cerebral IR injury. TRPC6: transient receptor potential canonical channel 6; Ca^2+^: calcium; CAMKIV: Ca^2+^/calmodulin-dependent kinase IV; CREB: cAMP-response element binding protein; NF-κB: nuclear factor (NF)-κB.

During the process of ischemic stroke, increasing evidences support that TRPC6 seems to be a protective pathway. In comparison with TRPC1/3/4/5, TRPC6 is significantly degraded in neurons following exposure to ischemia, resulting in ischemic neuronal death. The maintenance of TRPC6 expression via CREB-dependent mechanisms has a neuroprotective effect, and has potential as a protective strategy against ischemic stroke (Du et al., 2010). In addition, TRPC6 suppresses NMDAR-induced Ca^2+^ elevation and neuronal excitotoxicity, and protects neurons from ischemic brain injury (Li et al., 2012). Resveratrol, neuroprotectin D1, and the main compound of green tea, (−)-epigallocatechin-3-gallate, inhibit calpain-mediated TRPC6 proteolysis and activate MEK/ERK or CaMKIV-dependent CREB pathways, therefore improving neurological status in experimental stroke (Lin et al., 2013a; Yao et al., 2013, 2014). Particularly, the TRPC6 activator, hyperforin, also contributes to neuroprotection after ischemic stroke by blocking TRPC6 degradation accompanied by elevation of phosphorylated CREB in CaMKIV and Ras/MEK/ERK-dependent mechanisms (Lin et al., 2013b). Furthermore, calycosin increases neuronal TRPC6 and phosphorylated CREB expression in response to brain ischemic damage (Guo et al., 2017). We believe that the TRPC6-CaMKIV-CREB pathway may be an important mechanism of ischemic stroke (Figure 2). There is increasing research interest in stem cell therapy for ischemic stroke. Animal researches have indicated that bone marrow-derived stromal cells (BMSCs) improve outcomes in stroke, although the regulatory mechanisms have yet to be clarified (Yang et al., 2014). Surprisingly, TRPC6 channels have been shown to participate in the neuroprotective effect of BMSCs and oxiracetam combination therapies in acute brain IR damage (Wang et al., 2019). Furthermore, overexpression of TRPC6 in BMSCs improves neuronal functions in rats after ischemic stroke, which is associated with BDNF/CREB pathway (Li W. et al., 2019). The inflammatory response plays a critical role in the pathophysiology of ischemic stroke. The proinflammatory cytokine, interleukin (IL)-17A, was shown to be linked with brain IR injury (Gelderblom et al., 2012). Another study suggested that TRPC6 may act downstream of IL-17A, and indicated that IL-17A could promote degradation of TRPC6, thus exacerbating cerebral IR injury (Zhang et al., 2014). Taken together, the evidences outlined above indicate that TRPC6 has a positive function in neuroprotection and that TRPC6 expression is reduced during ischemic stroke. However, it should be noted that TRPC6 may also have harmful effects on neurons following cerebral IR injury. In contrast to previous studies, TRPC6 expression was proved to be increased in wild-type mice neurons after ischemic stroke. Increased TRPC6 levels or overexpression of TRPC6 exacerbated IR injury-induced infarct volume, Ca^2+^ elevation, and neuronal death (Chen J. et al., 2017). Deletion of TRPC3/6/7 was shown to decrease activation of the proapoptotic factor and nuclear factor (NF)-κB, attenuate NF-κB nuclear translocation, and enhance activation of PKB, finally leading to resistance against cerebral IR injury (Chen X. et al., 2017). The causes for these discrepancies among studies remain unclear; however, they might have been due to species differences, distinct animal ischemic stroke models, dissimilar experimental environments, and researchers. Therefore, more studies are required to clarify the specific function of the TRPC6 channel in ischemic stroke.

The evidence discussed here indicates that compounds targeting TRPC6 may have clinical potential as novel agents for prevention and treatment of ischemic stroke. It should be noted that interventions for most of the experiments mentioned above are performed under intracerebroventricular injection or *in vitro* cell condition. However, taking into consideration that TRPC6 is broadly distributed in human tissues (Riccio et al., 2002), both agonists and antagonists of TRPC6 might bring some systematic adverse effects. Besides, hyperforin had been shown to induce TRPC6-dependent Na^+^ currents and entry and subsequently decrease the Na^+^ gradients across the membrane thus may inhibit the neuronal amine uptake (Leuner et al., 2007; Wang et al., 2020). Furthermore, hyperforin could also function as a protonophore and generate H^+^ currents in a TRPC6-independent way in several cells (cortical microglial cells, chromaffin cells, lipid bilayers, and HEK-293 cells), and thereby suppress the neurotransmitter uptake through sodium-proton exchangers (Sell et al., 2014). Thus, many factors should be thoroughly considered before studies are conducted to explore the effect TRPC6 on ischemic stroke.

## Conclusion and Perspective

Due to its high prevalence adjuvant and novel treatments for ischemic stroke are all under active investigation; e.g., digital therapeutics, microRNA-based therapeutics, cell therapy, and exosome therapy (Sun et al., 2018; Venkat et al., 2018a, b; Bayraktutan, 2019; Choi et al., 2019). Despite advances in therapy, stroke remains a major health issue. The studies discussed in this review suggest that investigation of the TRPC and TRPC6 channels may hold promise for the development of novel directions in ischemic stroke therapies. We have emphasized some important clues regarding the involved mechanisms of TRPC6 in physiology and pathophysiology of cerebral ischemia. More data and solid evidence of TRPCs and TRPC6 in stroke therapy are urgently needed to bridge the gap between current pre-clinical medical researches and clinical practice. Further studies are required to support the speculations regarding the promising therapeutic roles of TRPCs and TRPC6 in ischemic stroke, and to determine the mechanisms underlying these roles.

## Author Contributions

LL wrote this manuscript. SZ and XX designed the general idea. All authors edited the drafts of the manuscript.

## Conflict of Interest

The authors declare that the research was conducted in the absence of any commercial or financial relationships that could be construed as a potential conflict of interest.
